# Development and Preliminary Validation of the KOOS-ACL: A Short Form Version of the KOOS for Young Active Patients With ACL Tears

**DOI:** 10.1177/03635465231160728

**Published:** 2023-04-07

**Authors:** Hana Marmura, Paul F. Tremblay, Alan M.J. Getgood, Dianne M. Bryant

**Affiliations:** * Faculty of Health Sciences, Western University, London, ON, Canada; † Fowler Kennedy Sport Medicine Clinic, London, ON, Canada; ‡ Bone and Joint Institute, Western University, London, ON, Canada; § Lawson Research, London Health Sciences Centre, London, ON, Canada; ‖ Department of Psychology, Western University, London, ON, Canada; ¶ Schulich School of Medicine and Dentistry, Western University, London, ON, Canada; # Department of Health Research Methods, Evidence and Impact, McMaster University, Hamilton, ON, Canada; Investigation performed at Western University, London, Ontario, Canada

**Keywords:** knee ligaments, ACL, patient-reported outcome, validation, KOOS, young athletes

## Abstract

**Background:**

The Knee injury and Osteoarthritis Outcomes Score (KOOS) is a widely used region-specific outcome measure for assessing patients of all ages with a variety of knee conditions. Use of the KOOS for young active patients with anterior cruciate ligament (ACL) tear has been called into question regarding its relevance and interpretability for this specific population. Furthermore, the KOOS does not have adequate structural validity for use in high-functioning patients with ACL deficiency.

**Purpose:**

To develop a condition-specific short form version of the KOOS that is appropriate for the young active population with ACL deficiency: the KOOS-ACL.

**Study Design:**

Cohort study (diagnosis); Level of evidence, 2.

**Methods:**

A baseline data set of 618 young patients (≤25 years old) with ACL tears was divided into development and validation samples. Exploratory factor analyses were conducted in the development sample to identify the underlying factor structure and to reduce the number of items based on statistical and conceptual indicators. Confirmatory factor analyses were conducted to check fit indices of the proposed KOOS-ACL model in both samples. Psychometric properties of the KOOS-ACL were assessed using the same data set, expanded to include patient data from 5 time points (baseline and postoperative 3, 6, 12, and 24 months). Internal consistency reliability, structural validity, convergent validity, responsiveness to change, floor/ceiling effects, and detection of treatment effects between surgical interventions (ACL reconstruction alone vs ACL reconstruction + lateral extra-articular tenodesis) were assessed.

**Results:**

A 2-factor structure was deemed most appropriate for the KOOS-ACL. Of 42 items, 30 were removed from the full-length KOOS. The final KOOS-ACL model showed acceptable internal consistency reliability (α = .79-.90), structural validity (comparative fit index and Tucker-Lewis index = 0.98-0.99; root mean square error of approximation and standardized root mean square residual = 0.04-0.07), convergent validity (Spearman correlation with International Knee Documentation Committee subjective knee form = 0.61-0.83), and responsiveness across time (significant small to large effects; *P* < .05).

**Conclusion:**

The new KOOS-ACL questionnaire contains 12 items and 2 subscales—Function (8 items) and Sport (4 items)—relevant to young active patients with an ACL tear. Use of this short form would reduce patient burden by more than two-thirds; it provides improved structural validity as compared with the full-length KOOS for our population of interest; and it demonstrates adequate psychometric properties in our sample of young active patients undergoing ACL reconstruction.

There is wide variation in the outcome measures used to assess patients with anterior cruciate ligament (ACL) injuries and reconstructions.^20^ One of the most commonly used patient-reported outcome measures for ACL injury is the Knee injury and Osteoarthritis Outcome Score (KOOS).^20^ The KOOS was developed as a knee-specific outcome measure intended to capture the severity of knee-related symptoms after a knee injury and throughout the development of knee osteoarthritis.^15,20^ The KOOS consists of 42 items grouped into 5 subscales or domains: Symptoms, Pain, Activities of Daily Living (ADL), Sport and Recreation function (Sport/Rec), and Quality of Life (QOL).^17^

The utility of the KOOS for patients with ACL tears has recently been called into question.^5,20,26^ The KOOS is a region-specific (knee) measure not tailored to patients with ACL injuries. Although studies have reported adequate reliability of the KOOS in patients with ACL deficiency,^19,22^ many of its questions have been critiqued for being “too easy” for high-functioning individuals, resulting in ceiling effects that make detecting differences among patients more difficult.^20^ Additionally, the KOOS does not address knee stability, a hallmark of ACL injury, recovery, and reconstruction success.^20^ At 42 items, the KOOS is quite lengthy and may increase patient burden, especially when included in a battery of outcome measures and clinical testing. Despite the known limitations, the KOOS continues to be a consistently used patient-reported outcome measure in ACL research.

A significant gap in the psychometric evaluation of orthopaedic outcome measures is a lack of structural validity assessment. Structural validity is defined as “the degree to which the scores of a patient-reported outcome measure are an adequate reflection of the dimensionality of the construct to be measured.”^10^ Essentially, structural validity is a measure of how well individual questionnaire items group to represent a unidimensional concept or distinct domain. Structural validity should be assessed for all multi-item outcome measures; it provides valuable information on the acceptability of outcome subscales.^10^

In a previous set of analyses, Marmura et al^9^ determined that the intended structure of the KOOS had poor structural validity in a sample of >600 young patients with ACL deficiency. This led us to conclude that the 5 subscale scores obtained from the KOOS for patients with ACL tears were probably not well representing the hypothesized constructs, with significant overlap among the Symptoms, Pain, and ADL domains.^9^ The KOOS contains many important questions related to patients’ experiences with a knee injury and osteoarthritis, but a shorter scale that addresses the issues of structural validity may provide similar or improved relevance and interpretability of patients’ experience while reducing responder burden.

Therefore, the purpose of this study was to develop and validate a disease-specific short form version of the KOOS appropriate for the young active population with ACL deficiency: the KOOS-ACL. It was hypothesized that a shorter version of the KOOS with adequate structural validity and adequate psychometric properties could be constructed using factor analysis.

All abbreviations are defined in Table 1.

**Table 1:** Abbreviations Used

ACL	anterior cruciate ligament
ACLR	anterior cruciate ligament reconstruction
ADL	Activities of Daily Living
ANOVA	analysis of variance
CFA	confirmatory factor analysis
CFI	comparative fit index
EFA	exploratory factor analysis
IKDC	International Knee Documentation Committee subjective knee form
KOOS	Knee injury and Osteoarthritis Outcome Score
KOOS-12	12-item Knee injury and Osteoarthritis Outcome Score
KOOS-ACL	Knee injury and Osteoarthritis Outcome Score–Anterior Cruciate Ligament
KOOS JR	Knee injury and Osteoarthritis Outcome Score Joint Replacement
LET	lateral extra-articular tenodesis
QOL	Quality of Life
RMSEA	root mean square error of approximation
SRMR	standardized root mean square residual
TLI	Tucker-Lewis index
Tukey HSD test	Tukey honestly significant difference test

## Methods

### Sample

The data set used to develop the KOOS-ACL was obtained during the Stability 1 study, a randomized clinical trial of young active patients undergoing primary ACL reconstruction (ACLR).^4^ This study was approved by the Western University Health Sciences Research Ethics Board (No. 104524). Patients were randomized to receive a hamstring tendon autograft ACLR, either alone or with a lateral extra-articular tenodesis. Inclusion criteria for the trial were patients aged ≤25 years who were deemed at high risk of surgical failure and reinjury based on meeting ≥2 of the following criteria: a pivot-shift grade ≥2, a desire to return to high-risk/pivoting sports, and generalized ligamentous laxity.^4^ A detailed study protocol and trial results are available elsewhere.^3,4^

The sample consisted of 618 patients recruited from 9 centers, 7 in Canada and 2 in Europe, from March 2014 to March 2017. Complete KOOS data were available for 606 patients (98%). Baseline data (postinjury, preoperative) were used during scale development and divided into development and validation samples. The full data set of patients across 5 time points (baseline and postoperative 3, 6, 12, and 24 months) was used for validation of psychometric properties.

### Outcome Measure

The KOOS questionnaire includes 5 subscales—Symptoms (7 items), Pain (9 items), ADL (17 items), Sport/Rec function (5 items), and QOL (4 items)—each with 5 possible categorial responses; however, these response options are not the same across all 42 items. The subscale scores are created separately by summing the score on each item (0-4) and then linearly transforming the sum to a score from 0 to 100 (worst to best), where 0 represents extreme knee problems and 100 represents no knee problems.^16^ The developers indicated that a composite score can be created by averaging the desired number of subscale scores. Yet, a total score should never be utilized for the KOOS as the ADL scale would inappropriately influence the total, given its higher number of items.^14^

### Factor Structure

An exploratory factor analysis (EFA) was used to determine the number of factors (subscales) that would be most appropriate for a short form ACL-specific scale. EFA is a statistical method used to examine the underlying structure of the correlations among variables (eg, items) in a data set by investigating how items group and form latent factors. Latent factors are constructs not directly measured but inferred by the correlations of conceptually related variables. In the case of the KOOS, the 5 subscale domains imply that the latent factors are symptoms, pain, ADL, sport/rec, and QOL. Loadings are standardized regression coefficients representing the correlation between questionnaire items and latent factors. Target loadings indicate how strongly an item loads onto its intended factor, while cross-loadings indicate how an item loads on the other latent factors in a model. A good item will have a high target loading and low cross-loadings, indicating little overlap across domains. It is expected that subscale domains may correlate, and this is modeled by including an oblique rotation in the EFA.

### Item Reduction

Once the appropriate number of latent factors for the KOOS-ACL was identified using EFA, it was possible to evaluate the quality of the items and remove those that were not contributing conceptually or statistically to each latent variable in 2 stages. In stage 1, items that did not load substantially on any latent factor (<0.40) or that loaded substantially on >1 factor (cross-loadings >0.2) and thus exhibited poor discriminant validity were eliminated one at a time to reduce redundancy among the subscales.^24^ In stage 2, items were removed for repetitive content. On any given factor, when 2 items were judged to ask a nearly identical or very similar question, the items with the lowest target loading were removed.

### Structural Validity

A confirmatory factor analysis (CFA) using an ordinal specification procedure was conducted on the resultant set of items for the KOOS-ACL in the development sample (half the baseline data set) to obtain fit indices in the simple structure of the model, which would be utilized in clinical practice (no cross-loadings). CFA is a statistical method similar to EFA; however, instead of identifying the underlying structure of a particular data set, it is used to confirm or discredit a preexisting or proposed structure. Model fit was assessed using the chi-square test statistic (χ^2^), root mean square error of approximation (RMSEA) with 90% CI, comparative fit index (CFI), Tucker-Lewis index (TLI), and standardized root mean square residual (SRMR). The robust estimates of all indices were reported. Adequate fit was defined as CFI and TLI >0.95 and RMSEA and SRMR <0.08. Chi-square and the associated *P* value were not used to formally assess fit, because χ^2^ is sensitive to sample size and likely to reject the null hypothesis of good fit in any sample >200 participants. CFA was then used to test the structural validity of the KOOS-ACL in the validation sample.

### Preliminary Validation of Psychometric Properties

Internal consistency reliability, structural validity, convergent validity, internal responsiveness, floor/ceiling effects, and detection of treatment effects were assessed testing our hypotheses and using the statistical methods and thresholds outlined in Table 2. These properties were assessed at each of the 5 study time points (baseline and postoperative 3, 6, 12, and 24 months). All analyses were conducted in R Studio using the *lavaan*, *effsize*, *dplyr*, and *psych* software packages.^13,18,23,25^

**Table 2:** Definitions, Hypotheses Being Tested, Statistical Methods, and Acceptable Thresholds Used to Investigate Psychometric Properties of the KOOS-ACL for Validation^*a*^

Psychometric Property	Definition^10^ and Hypotheses	Statistical Method	Acceptable Threshold
Internal consistency reliability	The degree of interrelatedness between items representing 1 construct Hypothesis: strong internal consistency reliability of both subscales, indicating unidimensionality	Cronbach alpha (α)^21^	.70 < α < .90
Structural validity: measurement invariance across time	The degree to which scores of an outcome measure adequately reflect the dimensionality of the intended constructs measured; a type of construct validity Equal form: the same factor structure is present across time Equal loadings: the same relationships between items and their factors are present across time Equal intercepts: the predicted scores of items can vary across time but at a constant level of the construct Hypothesis: adequate fit indices and change in fit indices reflecting structural validity and measurement invariance from baseline to postoperative 24 mo	Confirmatory factor analysis with analysis of fit indices	CFI and TLI ≥0.95 RMSEA and SRMR^6^≤0.08 Change in CFI <0.005 and change in RMSEA <0.01 with incremental model constraints
Convergent validity	The degree to which the relationship with another outcome measure intended to measure the same constructs is as hypothesized; a type of construct validity Hypothesis: moderate to strong correlation of subscale and composite scores with the IKDC, equivalent to correlations seen between the full-length KOOS and IKDC; strong correlation with full-length KOOS	Spearman correlation (ρ) with IKDC (owing to nonnormal distribution of scores at some time points)	ρ > 0.60 with IKDC^11^ ρ > 0.70 with KOOS *P* < .05
Responsiveness to change across time	The ability of an outcome measure to detect expected change over time in the intended constructs being measured Hypothesis: significant improvement in Sport scores from baseline to postoperative 24 mo and in Function scores from baseline to postoperative 12 mo; strong correlation between change scores of the KOOS-ACL and full-length KOOS	Paired *t* tests of subscale scores between time points, with Cohen *d* effect size^23^ Spearman correlation (ρ) between KOOS-ACL and full-length KOOS score mean differences across time These analyses were conducted assuming measurement invariance across time.	*d* > 0.2 *P* < .05 ρ > 0.70 with KOOS mean differences^11^ *P* < .05
Floor and ceiling effects	The proportion of the respondent distribution that will score on the extremes of an outcome measure Hypothesis: no significant ceiling effects in Sport scores from baseline to postoperative 24 mo or in Function scores from baseline to postoperative 12 mo	Percentage of patients scoring 0 (floor) and 100 (ceiling) points^7^	<15% scores = 100 or 0
Detection of treatment effects	The ability of a measure to detect a treatment effect between groups hypothesized to produce different outcomes Hypothesis: significant differences in subscale scores with minimally small effect sizes between patients who had ACLR alone vs those who had ACLR + LET at postoperative 12 and 24 mo; a significant time × group interaction between patients who had ACLR alone and those who had the additional LET procedure	ANOVA between groups (ACLR alone vs ACLR + LET), Tukey HSD test for multiple comparisons, Cohen *d* effect sizes^23^ Mixed ANOVA of repeated measures (group = between-patients factor, time = within-patient factor)	*d* > 0.2 *P* < 0.05

a ACLR, anterior cruciate ligament reconstruction; ANOVA, analysis of variance; CFI, comparative fit index; HSD, honestly significant difference; IKDC, International Knee Documentation Committee subjective knee form; KOOS, Knee injury and Osteoarthritis Outcome Score; KOOS-ACL, Knee injury and Osteoarthritis Outcome Score–Anterior Cruciate Ligament; LET, lateral extra-articular tenodesis; RMSEA, root mean square error of approximation; SRMR, standardized root mean square residual; TLI, Tucker-Lewis index.

## Results

### Sample

Complete baseline KOOS data required for short-form development was available for 606 patients (98%) with 2% missing data (Table 3). Baseline KOOS item descriptive statistics are available in Appendix 1 (see Table A1, available in the online version of this article).

**Table 3:** Patient Characteristics^*a*^

	No. (%) or Mean ± SD
Sex	
Male	294 (48.5)
Female	312 (51.5)
Age, y	19.0 ± 3.2
Sport participation	
Soccer	311 (51)
Running	208 (34)
Basketball	175 (29)
Volleyball	134 (22)
Hockey	106 (17)
Downhill skiing	96 (16)
Football	83 (14)
Rugby	78 (13)
Baseball/softball	64 (11)
Skating	44 (7)
Tennis	44 (7)
Other	234 (39)
Graft	
ACLR alone (all hamstring tendon autograft)	306 (50.5)
ACLR + LET	300 (49.5)

a ACLR, anterior cruciate ligament reconstruction; LET, lateral extra-articular tenodesis.

### Factor Structure

The initial EFA analyses revealed that a 2-factor model would be most appropriate for our data. When a 3-factor model was used, no item was most strongly correlated with the third factor. Between the 1- and 2-factor models, it was evident that a subset of items loaded more strongly onto a second factor rather than the first. Based on the pattern of loadings and question content, factor 1 was labeled “Function” (loading most strongly onto the Symptoms, Pain, and ADL items), and factor 2 was labeled “Sport” (loading most strongly onto the Sport/Rec and QOL items) (see Appendix 1, Table A2, available online).

### Item Reduction

Fifteen items were removed in stage 1 of item reduction based on the magnitude of target loadings and cross-loadings (see Appendix 1, Table A2, available online). This included 6 of 7 Symptoms items, 3 of 9 Pain items, 3 of 17 ADL items, 2 of 5 Sport/Rec items, and 1 of 4 QOL items. Item q3 (confidence in the knee) had a target loading below our 0.4 threshold (0.33) once all other items were removed; however, it was maintained in the model owing to conceptual relevance, a low cross-loading (0.01), and the need for >3 items per subscale for appropriate representation of a construct.

Twelve items were removed in stage 2 of item reduction based on repetitive content (see Appendix 1, Table A3, available online). This included the remaining 6 Pain items, 5 of 14 remaining ADL items, and 1 of 3 remaining QOL items. Item q1 (awareness of knee problem) was removed for a target loading of 0.29, which was deemed too low to be accepted as part of the model. Last, items a9 and a15 (difficulty putting on socks/stockings and difficulty getting on/off toilet, respectively) were removed because the content was not considered relevant to the young population with ACL deficiency based on expert opinion (orthopaedic surgeon and clinical researchers including AMJG, DMB, HM).

### Structural Validity

**Figure 1.**
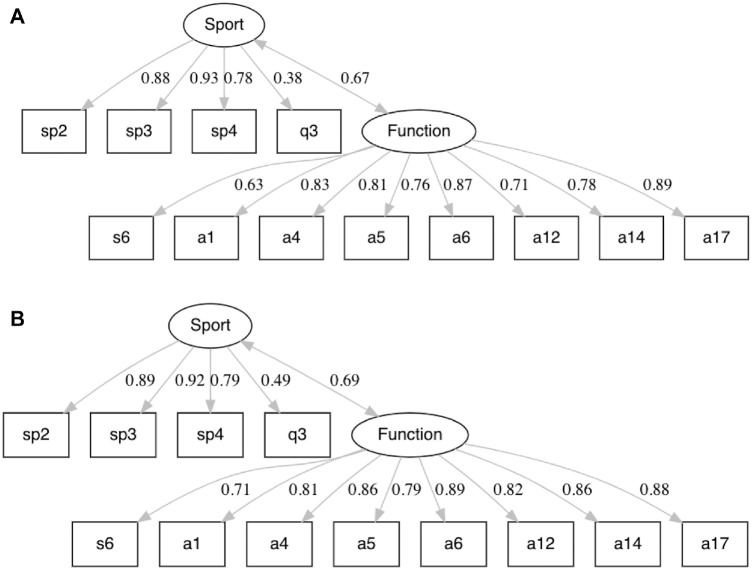
The KOOS-ACL model structure in the (A) development and (B) validation samples of young patients with ACL reconstructions, including standardized loadings and factor correlations. KOOS-ACL, Knee injury and Osteoarthritis Outcome Score–Anterior Cruciate Ligament.

After item reduction, a 2-factor model with 12 items remained as the short form version of the KOOS most appropriate for our patient population: the KOOS-ACL (Figure 1). The final structure contains 2 subscales: Function (8 items) and Sport (4 items). These would be reported as 2 separate scores, not a total score. As with the full-length KOOS, creating a total score would overrepresent the contribution of the Function scale and not be appropriate. A composite score can be calculated by averaging the Sport and Function scores. The fit indices of this model after CFA were acceptable without any modifications required: χ^2^(53) = 85.86 (*P* < .01), CFI = 0.99, TLI = 0.99, RMSEA = 0.05 (90% CI, 0.03-0.06), and SRMR = 0.04. The loadings on the Function factor ranged from 0.63 to 0.89, and the loadings on the Sport factor ranged from 0.38 to 0.93. The correlation between the factors was 0.67.

In the validation half of the baseline data set, no modifications of the proposed KOOS-ACL model were required to obtain acceptable fit. The CFA fit indices in the second half of the data set were as follows: χ^2^(53) = 116.83 (*P* < .01), CFI = 0.99, TLI = 0.99, RMSEA = 0.06 (90% CI, 0.05-0.08), and SRMR = 0.05. The loadings on the Function factor ranged from 0.71 to 0.89, and the loadings on the Sport factor ranged from 0.49 to 0.92. The correlation between the factors was 0.69. The loadings and correlations between the factors were quite similar in the development and validation samples (Figure 1, A and B, respectively).

The KOOS-ACL is available in Appendix 2, available online.

### Preliminary Validation of Psychometric Properties

Complete baseline and 3-, 6-, 12-, and 24-month postoperative KOOS data used for questionnaire validation were available for 606, 566, 570, 549, and 537 patients, respectively.

#### Internal Consistency Reliability

The KOOS-ACL Function and Sport subscales showed adequate internal consistency reliability among questionnaire items (0.7 < α < 0.95) (Table 4) at all 5 time points.

**Table 4:** Cronbach Alpha Values for the KOOS-ACL Subscales Used to Assess Internal Consistency Reliability^*a*^

	Baseline	3 mo	6 mo	12 mo	24 mo
Function	.90	.86	.83	.85	.89
Sport	.79	.84	.82	.88	.89

a KOOS-ACL, Knee injury and Osteoarthritis Outcome Score–Anterior Cruciate Ligament.

#### Structural Validity and Measurement Invariance Across Time

The KOOS-ACL model structure maintained adequate fit indices (CFI and TLI ≥0.95, RMSEA and SRMR ≤0.08) at all 5 time points when evaluated as individual models (Table 5) and as 1 model with repeated measures (Table 6), indicating equal form measurement invariance of the questionnaire across time (the basic 2-factor structure remained the same). The model was within acceptable thresholds of change in RMSEA (<0.01) but not CFI (<0.005) to confirm equal loadings or intercepts across time (Table 6).

**Table 5:** Fit Indices of the KOOS-ACL Structure at 5 Time Points Using Confirmatory Factor Analyses to Assess Structural Validity^*a*^

Time Point	χ^2^	CFI	TLI	RMSEA (90% CI)	SRMR
Baseline	138.57	0.99	0.99	0.05 (0.04-0.06)	0.04
3 mo	204.25	0.98	0.98	0.07 (0.06-0.08)	0.06
6 mo	121.29	0.99	0.98	0.04 (0.04-0.06)	0.05
12 mo	96.34	0.99	0.99	0.04 (0.03-0.05)	0.05
24 mo	108.24	0.99	0.99	0.04 (0.03-0.06)	0.04

a CFI, comparative fit index; KOOS-ACL, Knee injury and Osteoarthritis Outcome Score–Anterior Cruciate Ligament; RMSEA, root mean square error of approximation; SRMR, standardized root mean square residual; TLI, Tucker-Lewis index.

**Table 6:** Change in Fit Indices of the KOOS-ACL With Added Constraints Across All Time Points Using Confirmatory Factor Analysis to Assess Measurement Invariance^*a*^

	CFI (Change)	RMSEA (Change)
Equal form	0.935	0.035
Equal loadings	0.918 (–0.017)	0.039 (+0.004)
Equal intercepts	0.886 (–0.032)	0.046 (+0.007)

a CFI, comparative fit index; KOOS-ACL, Knee injury and Osteoarthritis Outcome Score–Anterior Cruciate Ligament; RMSEA, root mean square error of approximation.

#### Convergent Validity

Composite KOOS-ACL scores were strongly correlated with scores on the International Knee Documentation Committee (IKDC) subjective knee form at all 5 time points, indicating adequate construct validity (ρ > 0.70) (Table 7). Individual subscale scores showed weaker but significant moderate to strong correlations with IKDC scores (ρ > 0.60). These correlations were similar to correlations between scores on the full-length KOOS and IKDC. KOOS-ACL Function and Sport scores were strongly correlated with full-length KOOS ADL and Sport/Rec scores at all 5 time points (ρ = 0.78-0.96).

**Table 7:** KOOS-ACL and Full-Length KOOS Correlation With IKDC to Assess Convergent Validity^*a*^

Time Point	KOOS-ACL, ρ			KOOS, ρ		
	Composite Score	Function	Sport	Composite Score	Function	Sport
Baseline	0.82	0.76	0.72	0.86	0.77	0.76
3 mo	0.77	0.67	0.70	0.84	0.69	0.75
6 mo	0.80	0.64	0.77	0.84	0.69	0.79
12 mo	0.83	0.61	0.81	0.85	0.65	0.81
24 mo	0.82	0.64	0.80	0.87	0.67	0.80

a All correlations are presented as Spearman rank correlation coefficient (ρ) to assess nonnormally distributed data. All correlations were statistically significant with *P* < .05. IKDC, International Knee Documentation Committee subjective knee form; KOOS, Knee injury and Osteoarthritis Outcome Score; KOOS-ACL, Knee injury and Osteoarthritis Outcome Score–Anterior Cruciate Ligament.

#### Responsiveness

**Figure 2.**
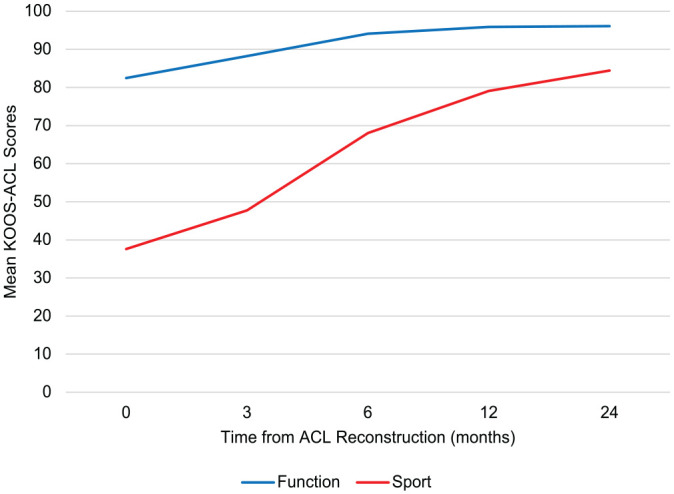
Mean KOOS-ACL scores from baseline to postoperative 2 years. ACL, anterior cruciate ligament; KOOS-ACL, Knee injury and Osteoarthritis Outcome Score–Anterior Cruciate Ligament.

The KOOS-ACL showed adequate ability to detect expected change over time, with a main effect of time on Function (*F* = 226.83; *P* < .01) and Sport (*F* = 658.58; *P* < .01) (Figure 2). Significant improvements with small to large effect sizes were seen in each subscale at incremental time points, except for the Function subscale and total score from 12 to 24 months (see Appendix 1, Table A4, available online). Change scores on the KOOS-ACL were highly correlated with those on the full-length KOOS.

#### Floor and Ceiling Effects

The KOOS-ACL showed significant ceiling effects (>15% of patients scoring 100) in the Function subscale after 6 months and in the Sport subscale after 12 months, similar to levels of the full-length KOOS (see Appendix 1, Figure A1, available online). Floor effects were well below the acceptable threshold for all scores and time points.

#### Detection of Treatment Effects

**Figure 3.**
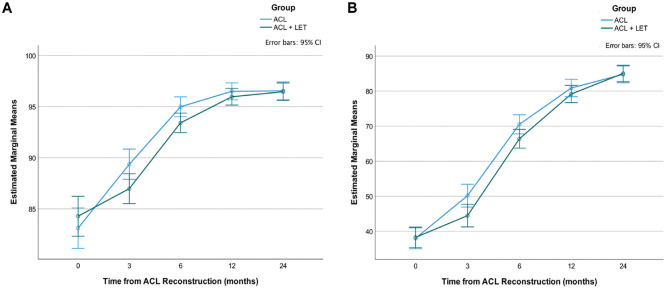
KOOS-ACL (A) Function and (B) Sport estimated marginal mean scores across time by group (ACL reconstruction alone vs the addition of a LET). ACL, anterior cruciate ligament; KOOS-ACL, Knee injury and Osteoarthritis Outcome Score–Anterior Cruciate Ligament; LET, lateral extra-articular tenodesis.

There were negligible effect sizes indicated by scores on the KOOS-ACL and full-length KOOS between patients who had ACLR alone (control group) and those who had ACLR with lateral extra-articular tenodesis (treatment group) (see Appendix 1, Table A5, available online). However, repeated-measures analyses of variance (ANOVAs) showed a significant interaction between time and group (ACLR alone vs ACLR + lateral extra-articular tenodesis) in both KOOS-ACL subscales: Function (*F* = 3.38; *P* = .02) and Sport (*F* = 2.83; *P* = .03) (Figure 3). This significant interaction was not detected for the full-length KOOS ADL (*F* = 2.77; *P* = .05) or Sport (*F* = 2.29; *P* = .07) subscale. The assumption of sphericity was violated for these analyses, so *P* values are reported with the Greenhouse-Geisser correction to reduce the likelihood of a type I error.

## Discussion

A short form version of the KOOS was designed for young active patients with ACL tears: the KOOS-ACL, containing 12 questions with 2 subscales representing the target constructs Function and Sport. The KOOS-ACL was based on statistical and conceptual indicators, focused on developing a questionnaire with adequate structural validity in the population of interest. Scoring of the KOOS-ACL to obtain a score out of 100, with higher scores indicating better outcomes, is done in the same way as the full-length KOOS (equivalent equations):

100−[(sumofsubscaleitems×100)/maximumsubscalescore]

100−[(averageofsubscaleitems/4)×100)].

The maximum scores for the Function and Sport subscales are 32 and 16, respectively. A total score should not be calculated using this method. A composite score could be calculated by averaging the 2 subscale scores, although psychometric properties of the composite score were not assessed.

The benefit of the KOOS-ACL over the original full-length KOOS for young active patients with ACL tears is 3-fold. First, this short form was developed in a large sample of young active patients undergoing ACLR, ensuring relevance to the population of interest. Second, the KOOS-ACL exhibits adequate structural validity in groups of patients from baseline to postoperative 2 years, using strict thresholds for fit indices.^6^ Structural validity of the full-length KOOS could not be confirmed in this population at baseline because the Pain, Symptoms, and ADL factors showed significant overlap and cross-construct relationships, meaning that it is unclear which of these 3 constructs each subscale score represents.^9^ Confirming structural validity allows for confidence when interpreting subscale scores—that subscale scores reflect the construct of interest (Function and Sport). Measurement invariance across time was confirmed by RMSEA but not CFI, as model fit changed beyond the acceptable threshold when loadings and/or intercepts were constrained to be equal across time. Therefore, even though the overall structure (items making up each subscale) is constant, the magnitude of the relationship between items and these constructs was not perfectly maintained across time points. Comparing 5 time points means that small differences can accumulate and have an incremental effect on measures of longitudinal invariance. Generally, our results support the structural validity of the KOOS-ACL model at individual follow-ups and across time. More sensitive analyses would be needed to investigate invariance and individual items/time points that may be problematic in specific samples.

Last, the KOOS-ACL will greatly reduce patient burden and the number of irrelevant items and will be practical to administer. This may help decrease the proportions of patients lost to follow-up in research or clinical care. These improvements have been made without losing adequacy of psychometric properties. The KOOS-ACL showed adequate internal consistency reliability, structural validity, convergent validity, and responsiveness based on widely accepted thresholds.

Additionally, researchers or clinicians may choose to administer the original KOOS and can then easily calculate KOOS-ACL scores from the applicable subset of items. One main difficulty with administering the KOOS to young patients with ACL tears appears related to the divergent trajectories/timelines of ACL injury or surgical recovery versus osteoarthritis development. In young active patients, the “easy” ADL items that may reveal osteoarthritis pathology in the long term (ie, postoperative 5-20 years for young patients with ACL tears) will often contribute to greater ceiling effects with little relevant differentiating information in the first 2 years of follow-up. In contrast, the more applicable sports-related items will provide important information within 2 years of injury or intervention but will be much less applicable at postoperative 15 or 20 years when patients’ lifestyles have shifted and the priority is to investigate signs and symptoms of osteoarthritis. Therefore, the same standardized patient-reported outcome measure may not be most appropriate for short- and long-term follow-up of ACL injury. It may be useful to administer the KOOS-ACL to high-functioning patients within the first 5 years of ACL injury, progressing to administering the full-length KOOS at later time points.

Some issues remain with our newly developed version of the KOOS. Ceiling effects remain a problem in the KOOS-ACL after about 6 months to 1 year. This could make statistical analyses and detection of treatment effects difficult and is a problem that requires further investigation. However, the maximum Function scores seen in approximately 50% to 60% of patients and the maximum Sport scores seen in 20% to 30% of patients by postoperative 1 to 2 years do reflect the expected positive outcomes of ACLR. Many young, active, and otherwise healthy patients should not have many limitations in function or sport by 2 years, and it may be reasonable to expect a significant percentage to score nearly perfect or perfect on this type of outcome. Last, the KOOS-ACL was not able to better detect treatment effects in this sample than the full-length KOOS, despite known statistically and clinically important differences in graft failure between the groups.^4^ This may be because of the relatively small number of adverse events in the sample at 2 years whereby the negative effect of reinjury/rupture on outcomes is washed out by the largely satisfactory outcomes in both groups. It is also possible that response shift influences these subjective outcomes or that simply no differences in these constructs exist between groups. The KOOS-ACL, not the full-length KOOS, detected a significant time × group interaction for each subscale, indicating a difference in the recovery trajectory between the groups as seen in the Stability 1 study primary outcome article, suggesting some increased sensitivity.^4^

Other short form versions of the KOOS have been developed, including the KOOS–Physical Function Short Form,^12^ KOOS Joint Replacement (KOOS JR),^8^ KOOS_global_,^7^ and KOOS-12.^2^ In development of the KOOS–Physical Function Short Form, only 1.7% of patients came from an ACL-specific data set with a mean age of 59 years (range, 27-89).^12^ As such, these patients were not representative of a young active population with an ACL tear. Similarly, the KOOS-12 and KOOS JR were developed and validated in patients undergoing total knee arthroplasty, which limits the ability to apply the questionnaires to patients with other knee injuries (eg, meniscal or ligamentous). The KOOS-12 developers suggest adding the remaining 4 “more difficult” Sport/Rec subscale items “for patients who aspire to a high-level function, such as those undergoing ACL evaluation.”^2^ This statement reiterates our belief that the sport-specific items are needed to assess the short-term changes in patient-reported outcomes in the young population with ACL tears.

Jacobs et al^7^ augmented the KOOS JR, adding 4 QOL items to create the KOOS_global_, in hopes of assessing patients specifically after ACLR and reducing ceiling effects. The KOOS_global_ showed convergent validity with the IKDC, responsiveness, and an 11% proportion of patients scoring 100 (ceiling effect; ie, <15% threshold).^7^ Although this version of the KOOS was created to assess patients after ACLR, it is not specific to addressing high-functioning patients as it is missing sport-related items relevant to this population, which are included in the KOOS-ACL. Furthermore, the structural validity of this measure is unknown.

### Limitations

Content validity, the degree to which an outcome measures the construct that it intends to, was not formally assessed in this study. Content validity of the full-length KOOS has been investigated, with patients with knee conditions and clinicians involved in item and subscale selection during development. A 2016 systematic review reported adequate content validity of the full-length KOOS in 4 studies, 2 of which were specific to patients with ACL injuries.^1^ The QOL and Sport/Rec subscales showed the highest content validity in younger patients and those with ACL injuries.^1^ However, a recent COSMIN (COnsensus-based Standards for the selection of health status Measurement Instruments) evaluation concluded that the KOOS had inadequate content validity based on target population and risk-of-bias issues with development and validity studies.^5^ The ADL items may not be relevant at all time points, but they allow for an investigation of improvement from acute to full recovery after ACLR. There are some gaps in the KOOS-ACL content (ie, no questions regarding knee stability), and this measure should be used in addition to other important patient-reported and clinical outcome measures to gain a complete picture of patient recovery. The shortened KOOS-ACL will likely fit better in such a battery of measures as compared with its full-length counterpart. The KOOS-ACL requires further psychometric testing and cross-validation before it can be recommended for clinical use. Fortunately, because the KOOS-ACL uses a subset of questions from the full-length KOOS, this model can be easily tested in other ACL data sets.

## Conclusion

The new KOOS-ACL questionnaire contains 12 items and 2 subscales relevant to young active patients with ACL tears: Function and Sport. The KOOS-ACL provides a valid shortened version of the KOOS with more relevant information and improved structural validity for the young active ACL population.

## Supplemental Material

sj-pdf-1-ajs-10.1177_03635465231160728 – Supplemental material for Development and Preliminary Validation of the KOOS-ACL: A Short Form Version of the KOOS for Young Active Patients With ACL Tears Click here for additional data file. Supplemental material, sj-pdf-1-ajs-10.1177_03635465231160728 for Development and Preliminary Validation of the KOOS-ACL: A Short Form Version of the KOOS for Young Active Patients With ACL Tears by Hana Marmura, Paul F. Tremblay, Alan M.J. Getgood and Dianne M. Bryant in The American Journal of Sports Medicine
