# Supplementary material for: Development and Preliminary Validation of the KOOS-ACL: A Short Form Version of the KOOS for Young Active Patients With ACL Tears
Source: Am J Sports Med. 2023 Apr 7;51(6):1447–56. doi: 10.1177/03635465231160728 (PMC10155282; doi:10.1177/03635465231160728)
Supplement: sj-pdf-1-ajs-10.1177_03635465231160728 – Supplemental material for Development and Preliminary Validation of the KOOS-ACL: A Short Form Version of the KOOS for Young Active Patients With ACL Tears [file sj-pdf-1-ajs-10.1177_03635465231160728.pdf]

Development and Preliminary Validation of the KOOS-ACL - A Short-form Version of the KOOS for Young Active Patients with ACL Tears

APPENDIX 1: Supplemental Tables and Figures

Table A1. Descriptive statistics of the Knee Injury and Osteoarthritis Outcome Score (KOOS) in the complete baseline dataset of 606 young patients with anterior cruciate ligament tears.

	Response Option								
**Item**	**0**	**1**	**2**	**3**	**4**	**Count**	**Mean**	**SD**	**Median**
s1	149	204	150	62	42	607	1.41	1.16	1
s2	118	140	159	142	48	607	1.77	1.23	2
s3	215	182	149	47	14	607	1.12	1.05	1
s4	299	145	57	39	67	607	1.06	1.36	1
s5	204	153	84	70	96	607	1.51	1.45	1
s6	202	225	145	28	6	606	1.03	0.92	1
s7	164	234	151	53	4	606	1.17	0.95	1
p1	55	100	222	203	26	606	2.07	1.02	2
p2	51	125	168	179	82	605	2.19	1.16	2
p3	263	174	114	45	10	606	0.95	1.03	1
p4	158	181	157	78	32	606	1.41	1.16	1
p5	358	178	52	14	4	606	0.56	0.80	0
p6	192	229	133	42	10	606	1.09	0.98	1
p7	403	134	57	10	2	606	0.47	0.76	0
p8	380	168	50	7	1	606	0.48	0.71	0
p9	267	225	96	16	2	606	0.78	0.83	1
a1	258	212	102	25	9	606	0.87	0.94	1
a2	251	208	118	20	9	606	0.89	0.93	1
a3	296	201	86	21	2	606	0.73	0.85	1
a4	311	197	79	17	2	606	0.68	0.83	0
a5	184	231	143	37	11	606	1.11	0.97	1
a6	403	140	52	8	3	606	0.46	0.75	0
a7	303	198	85	16	4	606	0.71	0.85	0.5
a8	277	196	97	25	11	606	0.84	0.96	1
a9	381	157	53	11	4	606	0.51	0.78	0
a10	405	151	43	5	2	606	0.43	0.69	0
a11	341	185	65	12	3	606	0.60	0.80	0
a12	356	170	56	22	2	606	0.59	0.83	0
a13	351	174	63	15	3	606	0.59	0.81	0
a14	424	135	42	4	1	606	0.39	0.66	0
a15	436	124	38	6	2	606	0.37	0.68	0
a16	121	191	193	72	29	606	1.50	1.08	1
a17	381	169	49	3	4	606	0.48	0.72	0
sp1	73	192	186	101	54	606	1.79	1.13	2
sp2	59	110	172	152	113	606	2.25	1.23	2
sp3	52	89	159	160	146	606	2.43	1.24	3
sp4	24	57	140	194	191	606	2.78	1.11	3
sp5	51	127	210	138	80	606	2.11	1.14	2
q1	7	15	66	302	216	606	3.16	0.80	3
q2	5	54	160	171	216	606	2.89	1.02	3
q3	13	82	200	191	120	606	2.53	1.02	3
q4	9	138	282	137	40	606	2.10	0.88	2

Note. For all items 0 represents the best knee function and 4 represents the best knee function.
SD = standard deviation

Table A2. Standardized factor loadings of two exploratory factor analyses of the Knee Injury and Osteoarthritis Outcome Score (KOOS) in young patients with anterior cruciate ligament tears.

			Stage 1 item Reduction	
	Factor 1	Factor 2	Factor 1	Factor 2
**Symptoms**				
s1	0.25	0.29		
s2	0.04	0.32		
s3	0.13	0.36		
s4	0.03	0.36		
s5*	0.01	0.46		
s6	0.46	0.22	0.50	0.14
s7	0.34	0.36		
**Pain**				
p1	0.26	0.5		
p2	0	0.69	0.05	0.65
p3	0.37	0.29		
p4*	0.17	0.42		
p5	0.75	-0.07	0.72	-0.03
p6	0.59	0.19	0.59	0.19
p7	0.53	0.07	0.53	0.08
p8	0.74	-0.15	0.72	-0.13
p9	0.67	0.08	0.67	0.08
**Activities of Daily Living**				
a1	0.63	0.19	0.62	0.20
a2	0.59	0.22		
a3	0.68	0.14	0.69	0.13
a4	0.7	0.06	0.69	0.07
a5	0.58	0.18	0.61	0.12
a6	0.79	-0.04	0.76	0.02
a7	0.68	0.1	0.70	0.09
a8	0.57	0.22		
a9	0.72	-0.09	0.74	-0.10
a10	0.79	-0.07	0.78	-0.06
a11	0.66	0	0.68	-0.02
a12	0.64	0.06	0.66	0.04
a13	0.7	0.01	0.71	0.00
a14	0.76	-0.09	0.74	-0.08
a15	0.85	-0.13	0.85	-0.12
a16	0.41	0.45		
a17	0.78	0.02	0.77	0.04
**Sport and Recreation**				
sp1	0.25	0.48		
sp2	0.07	0.71	0.12	0.68
sp3	-0.01	0.8	0.05	0.79
sp4	-0.13	0.85	-0.08	0.87
sp5	0.23	0.57		
**Quality of Life**				
q1	0	0.46	0.05	0.41
q2	0	0.36		
q3	-0.13	0.51	-0.09	0.50
q4	0.14	0.62	0.18	0.60

Items were removed in stage one (grayed out) if their target (strongest) loading < 0.4, and/or their cross (weakest) loadings > 0.2.
* = items had acceptable loadings during the initial EFA but took on unacceptable target loadings < 0.4 and/or cross loadings > 0.2 as other items were removed incrementally.

Table A3. Groups of items deemed to contain repetitive content in the KOOS.

Pair/Group of Similar Items	Questions	Target Loading
p2	Amount of pain with twisting/pivoting on your knee	0.65
**sp4**	**Degree of difficulty twisting/pivoting on your knee**	**0.87**
p5	Amount of knee pain walking on a flat surface	0.72
**a6**	**Degree of difficulty walking on a flat surface**	**0.76**
p6	Amount of knee pain going up or down stairs	0.59
**a1**	**Degree of difficulty descending stairs**	**0.62**
p7	Amount of knee pain at night while in bed	0.53
**a12**	**Degree of difficulty lying in bed (turning over, maintaining knee position)**	**0.66**
p8	Amount of knee pain sitting or lying	0.72
**a14**	**Degree of difficulty sitting**	**0.74**
p9	Amount of knee pain standing upright	0.67
**a4**	**Degree of difficulty standing**	**0.69**
**a9**	**Degree of difficulty putting on socks/stockings**	**0.74**
a11	Degree of difficulty taking off shoes/stockings	0.68
a3	Degree of difficulty rising from sitting	0.69
a7	Degree of difficulty getting in/out of car	0.7
a10	Degree of difficulty rising from bed	0.78
a13	Degree of difficulty getting in/out of bath	0.71
**a15**	**Degree of difficulty getting on/off toilet**	**0.85**
q4*	In general, how much difficulty do you have with your knee	0.60

Note: factor loadings are standardized
Items grouped in the same row represent similar question content.
Bolded items were maintained for the short-form KOOS based on higher target loadings among other items discussing similar content.
*Item q4 is not listed with other items but was found to overlap with all the ADL items and was removed based on a lower loading than the other items.

Table A4. Mean difference in KOOS-ACL scores across time and correlation to mean difference in full length KOOS scores across time, to assess responsiveness.

	Function	Sport
**Baseline to 3 months**		
Mean difference (95% CI)	5.39* (4.14 to 6.63)	10.44* (8.12 to 12.75)
Effect size (Cohen's d)	0.39 (small)	0.44 (small)
Correlation to KOOS mean difference	0.92	0.92
**3 to 6 months**		
Mean difference (95% CI)	5.81* (5.03 to 6.59)	20.19* (18.30 to 22.08)
Effect size (Cohen's d)	0.58 (medium)	0.87 (large)
Correlation to KOOS mean difference	0.85	0.91
**6 to 12 months**		
Mean difference (95% CI)	1.95* (1.35 to 2.55)	11.46* (9.79 to 13.12)
Effect size (Cohen's d)	0.28 (small)	0.57 (medium)
Correlation to KOOS mean difference	0.74	0.89
**12 to 24 months**		
Mean difference (95% CI)	0.25 (-0.43 to 0.93)	5.02* (3.26 to 6.77)
Effect size (Cohen's d)	0.03 (negligible)	0.26 (small)
Correlation to KOOS mean difference	0.76	0.83

* = significant difference in scores between time points ($p < 0.05$)

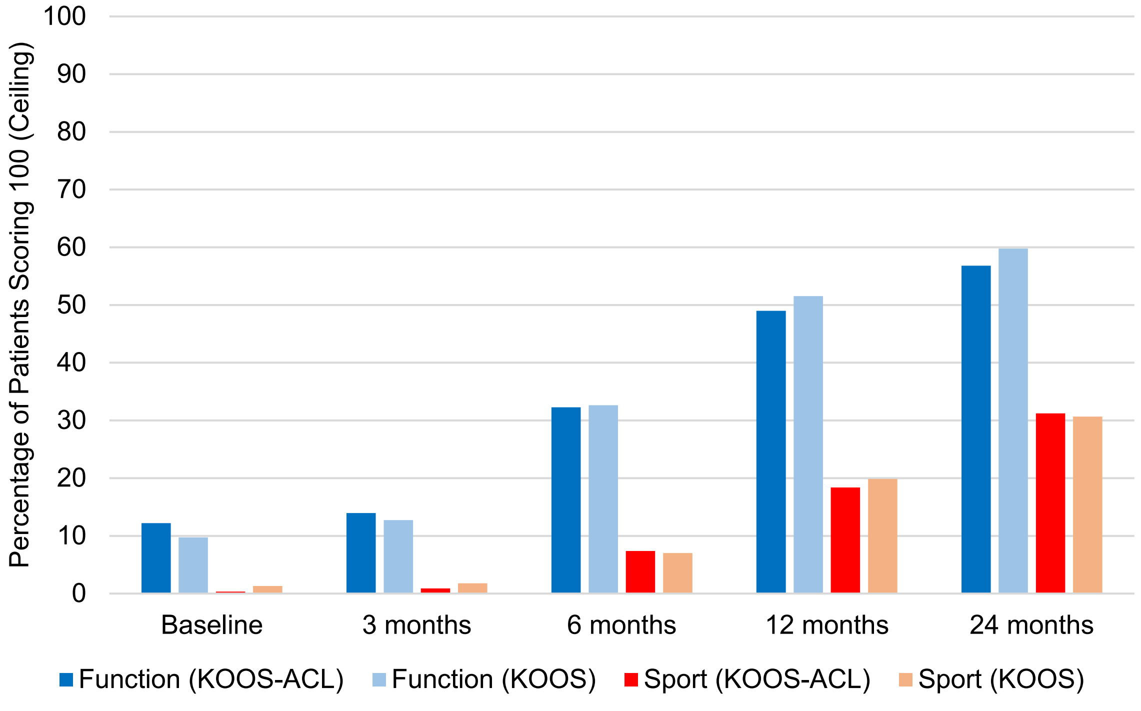

Figure A1. Ceiling Effects in KOOS-ACL and KOOS subscale scores from baseline to 2-years post-operative.

Table A5. Mean differences in KOOS-ACL and KOOS scores between treatment groups (ACL reconstruction alone versus ACL reconstruction plus lateral extra-articular tenodesis) with associated effect sizes, to assess detection of treatment effects.

	Function (KOOS-ACL)	Function (KOOS)	Sport (KOOS-ACL)	Sport (KOOS)
**12 Months**				
Mean difference	-0.68	-0.56	-1.94	-1.44
(95% CI)	(-1.84 to 0.49)	(-1.61 to 0.49)	(-5.26 to 1.38)	(-4.48 to 1.59)
Adjusted p-value	0.25	0.29	0.25	0.35
Effect Size	0.098 (trivial)	0.090 (trivial)	0.098 (trivial)	0.080 (trivial)
**24 Months**				
Mean difference	0.015	0.02	0.84	0.27
(95% CI)	(-1.30 to 1.33)	(-1.19 to 1.22)	(-2.34 to 4.00)	(-2.67 to 3.22)
Adjusted p-value	0.98	0.98	0.60	0.85
Effect Size	-0.0019 (trivial)	-0.0019 (trivial)	-0.045 (trivial)	-0.016 (trivial)

ANOVA analyses were performed, with Tukey's HSD test for multiple comparisons to obtain adjusted p-values. Cohen's d effect sizes are reported.

APPENDIX 2: **KOOS-ACL**

Instructions:
Answer every question by checking the appropriate box, only one box for each question. If you are unsure about how to answer a question, please give your best answer.

Function

1. How severe is your knee joint stiffness after first waking in the morning?

None	Mild	Moderate	Severe	Extreme

2 to 8. The following questions concern your physical function. By this we mean your ability to move around and to look after yourself. For each of the following activities, please indicate the degree of difficulty you have experienced in the last two weeks due to your knee.

	None	Mild	Moderate	Severe	Extreme
Descending Stairs					
Standing					
Bending to floor/picking up an object					
Walking on flat surface					
Lying in bed (rolling over, maintaining knee position)					
Sitting					
Light domestic duties (cooking, dusting, etc.)					

Sport

1 to 3. The following questions concern your physical function when being active on a higher level. The questions should be answered thinking of what degree of difficulty you have experienced during the last two weeks due to your knee.

	None	Mild	Moderate	Severe	Extreme
Running					
Jumping					
Twisting/pivoting on your knee					

4. How much are you troubled with lack of confidence in your knee?

Not At All	Mildly	Moderately	Severely	Totally

Scoring

Individual question items are scored as 0 to 4 (from “None” to “Extreme”, respectively (Function q1-8 and Sport q1-3), or “Not At All” to “Totally”, respectively (Sport q4)).

The KOOS-ACL Function and Sport scores can be calculated in the same way as full-length KOOS subscale scores.

The following equivalent equations can be used to calculate Function and Sport scores:

$$100 - \left(\frac{sum\ of\ subscale\ items * 100}{maximum\ subscale\ score}\right)$$

OR

$$100 - \left(\frac{average\ of\ subscale\ items}{4} * 100\right)$$

Function and sport scores should be calculated, assessed, and analyzed individually.

A total score should not be calculated.

A composite score can be calculated by averaging the two subscale scores, but comprehensive psychometric properties of this score have not been assessed.
